# Effects of Diets With Different Carbohydrate to Lipid Ratios on the Growth Performance, Ion Transport, and Carbohydrate, Lipid and Ammonia Metabolism of Nile Tilapia (*Oreochromis niloticus*) Under Long-Term Saline–Alkali Stress

**DOI:** 10.1155/2024/9388755

**Published:** 2024-11-14

**Authors:** Wei Liu, Erchao Li, Chang Xu, Liqiao Chen, Xiaodan Wang

**Affiliations:** ^1^Laboratory of Aquaculture Nutrition and Environmental Health, School of Life Sciences, East China Normal University, Shanghai 200241, China; ^2^Key Laboratory of Tropical Hydrobiology and Biotechnology of Hainan Province, Hainan Aquaculture Breeding Engineering Research Center, School of Marine Biology and Aquaculture, Hainan University, Haikou 570228, China

**Keywords:** ammonia metabolism, carbohydrate to lipid ratios, lipid metabolism, saline–alkali stress, tilapia

## Abstract

A 50-day test was adopted to compare the growth performance, liver histology, glucose metabolism, lipid (L) metabolism, ion transport, and ammonia metabolism of tilapia fed different carbohydrate–lipid (C:L) ratio diets under saline–alkaline water (salinity = 16 mmol/L and alkalinity = 35 mmol/L). The C and L levels of five isoenergetic (16.5 kJ/g) and isonitrogenous (32% protein) diets were C45%:L3% (L3), C38%:L6% (L6), C31%:L9% (L9), C24%:L12% (L12), and C17%:L15% (L15). This study found that the dietary C:L ratio did not affect the survival rate (SR), feed conversion ratio (FCR), or condition factor of tilapia in saline–alkali water, but fish in the L12 group had the highest weight gain (WG) rate and the lowest hepatosomatic index (HSI) compared with the other groups. Fish fed the higher C diet (L3 and L6) had a higher ion transport capacity and ammonia excretion capacity in gills. However, the highest mRNA expression of genes involved in glutamine metabolism and urea metabolism in the liver was found in the high-L diet groups (L12 and L15). In particular, a lower serum ammonia concentration was observed in the high-L diet groups (L12 and L15). In addition, biochemical indicators indicated that the L12 group had the highest liver pyruvic acid, lactic dehydrogenase (LDH), and lipase (LPS) and serum total cholesterol (T-CHO) contents. In summary, this study indicated that dietary Ls could promote glutamine metabolism and urea metabolism more than dietary Cs and then reduce the serum ammonia concentration of tilapia in saline–alkali water. A dietary C:L ratio of 2:1 was beneficial to the growth and ammonia excretion of tilapia in saline–alkali water in this study.

## 1. Introduction

Global food has an increasing demand for aquatic products [1]. Aquaculture is the most effective way to obtain enough aquatic products [2]. More notably, water resource shortages greatly limit the development of aquaculture [3]. The wide saline–alkali water area is an important way to solve the problem of water resource shortages in aquaculture [4, 5]. In addition, the exploitation and utilization of saline–alkali water resources is not only beneficial to the development of aquaculture but can also improve local economic benefits.

The stress of saline–alkali water on aquatic animals mainly consists of salinity stress and alkalinity stress [6]. As an environmental factor closely associated with osmotic pressure, changes in the salinity of the water environment will affect the physiological, biochemical, and metabolic activities of aquatic animals [7, 8], and long-term hypersaline exposure can lead to a low survival rate (SR), nutrient metabolism disorder, and reduced antioxidant capacity of aquatic animals [9, 10]. Alkalinity stress is primarily caused by high concentrations of CO_3_^2−^ and HCO_3_^−^ ions in the water [11], and its complex ionic composition can lead to acid–base imbalance and metabolic disorders in aquatic animals [12, 13] and eventually inhibit growth [14]. In addition, carbonate alkalinity can cause an ammonia metabolic burden in aquatic animals [15]. Various studies have shown that saline–alkali stress can cause respiratory disorders, disturb the antioxidant system, and even lead to death in aquatic animals [7, 16]. Previous studies have shown that more energy is used by aquatic animals to cope with stress caused by saline–alkali water, which greatly decreases the energy available for growth, resulting in poor growth performance [16]. Therefore, understanding the energy metabolism mechanism is crucial for the utilization of saline–alkali water resources.

As the three main nutrients, proteins, lipids (Ls), and carbohydrates (Cs) are also the main sources of energy for aquatic animals [17]. Protein plays a crucial role in the growth and development of fish [18]. However, studies have shown that higher dietary protein would increase the burden of ammonia metabolism in aquatic animals exposed to saline–alkali stress [19]. Both Ls and Cs have good protein-saving effects, which can effectively reduce the ammonia produced by protein catabolism [20, 21]. As an economical energy source [22], Cs serve as the direct energy source for aquatic animals to adapt to environmental stress [23]. Previous studies have shown that osmoregulation in aquatic animals expends a large amount of energy [24, 25], and salinity stress can increase the catabolism of Cs in the liver to meet more energy requirements [26]. Similarly, dietary Ls can improve some stress tolerance of fish by altering energy metabolism [27]. In addition, due to the better bioavailability of Ls compared to other nutrients in fish, Ls are more effective in conserving protein for growth than Cs [28]. Researches speculated that Ls are used for the energy supply of tilapia under high saline–alkali stresses [15]. More importantly, some studies have also found that saline–alkali stress can affect L metabolism in aquatic animals [16, 29]. However, until now, no reports of dietary Ls have been found in saline–alkali water. Therefore, it is of great significance to determine the appropriate C:L ratio in saline–alkali water as soon as possible.

Nile tilapia (*Oreochromis niloticus*) is a major farmed fish species because of its high nutrition, commercial value, and good tolerance against stress [30]. Hence, Nile tilapia has been widely used as a model organism in the study of saline–alkali mechanisms [15, 31]. In addition, as an omnivore fish, Nile tilapia can tolerate high C levels ranging from 38% to 46% and L levels ranging from 11% to 14% [32, 33]. Therefore, in this study, the effects of five different C:L ratios on growth performance, liver histology, C metabolism, L metabolism, ion transport, and ammonia metabolism were compared in Nile tilapia under saline–alkali stress. The results of this study clarified the energy metabolism and tolerance mechanism of Nile tilapia under saline–alkali stress and determined the optimal dietary C:L ratio, which can not only provide basic data for commercial farming of tilapia in saline–alkali water but also help the development of the corresponding feed industry.

## 2. Materials and Methods

Animal care and treatment procedures were conducted in strict accordance with the ethical requirements by the Animal Experiment of East China Normal University (permit number: E20120101).

### 2.1. Diets, Animals, and Experimental Design

Soybean oil and corn starch were utilized as dietary L and C sources, and the diets were made according to others' descriptions [34]. The diets varied in L and C composition: L3 (3% and 45%), L6 (6% and 38%), L9 (9% and 31%), L12 (12% and 24%), and L15 (15% and 17%). The detailed composition of the five diets is shown in Table 1. Nearly 800 juvenile Nile tilapia were sourced from a local tilapia company and acclimatized over a 2-week period in two 500-L buckets, during which they were fed a commercial diet. As part of the acclimation, the saline–alkali water conditions were gradually adjusted over the last 5 days, increasing salinity by approximately 3 mmol/L and alkalinity by approximately 7 mmol/L per day, culminating in levels of 16 ± 1 and 35 ± 1 mmol/L, respectively. Following acclimation, 375 healthy fish, averaging 1.19 ± 0.03 kg, were randomly distributed into 15 tanks (60 cm × 30 cm × 35 cm, 25 fish per tank), organized into five treatment groups with three replicates each. The five groups were, respectively, fed diets L3, L6, L9, L12, and L15. During the 50-day feeding trial, fish were fed daily at 08:00 and 16:00 until clear signs of satiation were observed, with daily food intake meticulously recorded. At 13:00 each day, water in each tank was exchanged using a siphon method, with the volume of water replaced amounting to 70% of the total volume in the aquaculture system. The tanks were maintained at a water temperature of 26 ± 2°C, ammonia was 0–0.2 mg/L, pH was 7.8–8.6, and a dissolved oxygen level exceeding 7.0 mg/L. Preparation of the saline–alkali water involved mixing NaHCO_3_, seawater, and dechlorinated tap water, which was then aerated for a day before use.

### 2.2. Sample Collection and Growth Performance

At the end of the 50-day trial, fish were fasted for 24 h and then anesthetized with MS-222 (40 mg/L) [36]. The length, weight, and number of fish from all tanks were recorded to calculate the SR, weight gain (WG) rate, feed conversion ratio (FCR), and condition faction (CF). Three fish per tank were randomly selected to collect whole blood by drawing tail vein blood using a sterile syringe. The blood was kept in a 4°C refrigerator overnight and centrifuged at 2500 rpm at 4°C for 10 min. The supernatant was taken and kept at −80°C for biochemical detection. The livers and gills of another 10 fish per tank were taken and immediately stored at −80°C. The livers were weighed to calculate the hepatosomatic index (HSI).

  $$\begin{array}{c} \text{Survival rate }\left(\text{SR},\%\right)=100\times \left[\frac{\text{Number of final fish}}{\text{Number of initial fish}}\right]. \end{array}$$

  $$\begin{array}{c} \text{Weight gain }\left(\text{WG},\%\right)=100\times \left[\frac{\text{Final body weight }\left(\text{g}\right)-\text{ initial body weight }\left(\text{g}\right)}{\text{Initial body weight }\left(\text{g}\right)}\right]. \end{array}$$

  $$\begin{array}{c} \text{Feed conversion ratio }\left(\text{FCR},\%\right)=100\times \frac{\text{ Feed consumption}}{\left(\text{Final biomass}-\text{initial biomass}+\text{ dead fish weight}\right)}. \end{array}$$

  $$\begin{array}{c} \text{Condition factor }\left(\text{CF},\text{ g}/{\text{cm}}^{3}\right)=100\times \frac{\text{Body weight }\left(\text{g}\right)}{{\left(\text{Body length }\left(\text{cm}\right)\right)}^{3}}. \end{array}$$

  $$\begin{array}{c} \text{Hepatosomatic index }\left(\text{HSI},\%\right)=100\times \left[\frac{\text{Wet liver weight }\left(\text{g}\right)}{\text{Wet body weight }\left(\text{g}\right)}\right]. \end{array}$$

### 2.3. Histological Analysis

Liver samples from three fish per group were fixed in 4% paraformaldehyde for 24 h, then dehydrated and cleared with ethanol, toluene, and xylene before embedding in paraffin. Sections of 5 µm thickness were cut using a Leica rm2125 rotary microtome (Leica, Germany), stained with hematoxylin and eosin (H&E), and examined under an Olympus BX51 microscope (Olympus, Tokyo, Japan). Quantitative measurements of liver cells were performed using Image-Pro Plus 6.0 software.

### 2.4. Biochemical Analysis

Two livers from each tank (*n* = 3) were accurately weighed, transferred to centrifuge tubes, added to a cold 0.86 saline solution (1:9, w/v), and then homogenized using a tissue grinder (Shanghai Jingxin, China; 60 Hz, 30 s). The homogenate was centrifuged at 3500 rpm at 4°C for 10 min, and the supernatant was collected and stored at −80°C. Liver homogenates were made to determine pyruvic acid contents and lactic dehydrogenase (LDH) and lipase (LPS) activities. The serum of three fish from each tank (*n* = 3) was used to detect glucose, total cholesterol (T-CHO), and triglyceride (TG). Livers and muscles of another three fish in each tank (*n* = 3) were taken to detect liver glycogen and muscle glycogen. All experimental procedures were performed according to the commercial kit instructions (Jiancheng, Ltd., Nanjing, China).

### 2.5. Quantitative Real-Time Polymerase Chain Reaction (PCR)

Two fish from each tank (*n* = 3) were randomly chosen for real-time PCR analysis. As per the manufacturer's instructions, TRIzol reagent (GLPBIO, USA) was used to extract total RNA from the liver and gills. The total RNA concentration was adjusted to 500 ng/*μ*L using a Nanodrop2000, and then reverse transcription was performed by a reverse transcription kit (Biosharp, China). The primers designed by Primer 5 software for quantitative polymerase chain reaction (qPCR) are shown in Table 2, and *β-actin* was used as the internal reference gene. An reverse transcription polymerase chain reaction (RT-PCR) kit was used for fluorescence quantification. The relative quantification of the target gene was estimated by using the 2^−*ΔΔCt*^ method [37].

### 2.6. Statistical Analysis

Statistical product and service solutions (SPSS) Statistics 26 (IBM, Armonk, NY, USA) was adopted for all statistical analyses. The data are expressed as the mean ± standard error of the mean (SEM). Differences among treatment groups were analyzed by one-way analysis of variance (ANOVA) followed by Duncan's multiple comparison test. *P* < 0.05 was regarded as statistically significant. Additionally, trends in the data were analyzed using orthogonal polynomial comparisons. The adjusted *R*^2^ (Adj. *R*^2^) was counted as previously described by the laboratory [38]. The association between WG and dietary L levels was assessed using quadratic broken line regression. Correlation network heatmap analysis was performed using https://www.omicshare.com.

## 3. Results

### 3.1. Growth and Physiological Parameters

**Figure 1:**
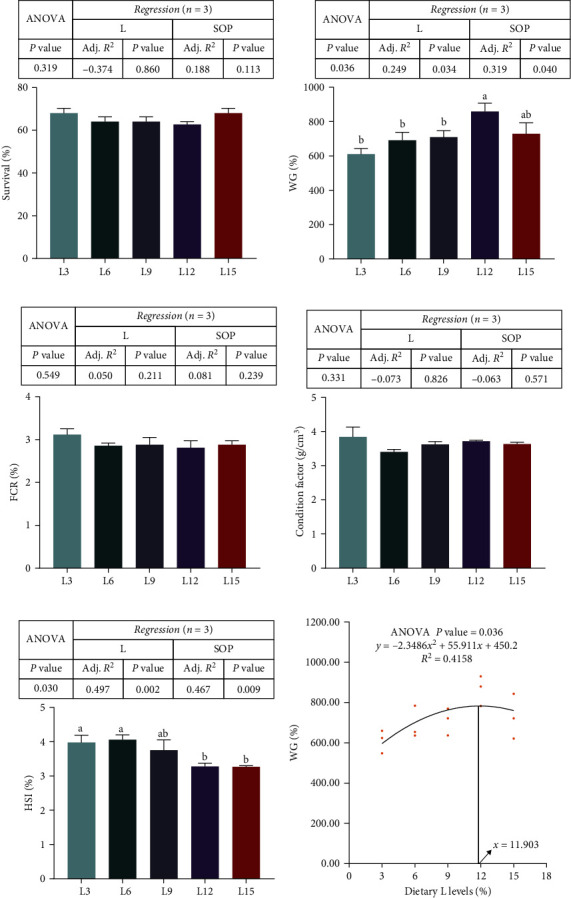
The effects of *O. niloticus* fed different diets on growth performance (survival (A), WG (B), FCR (C), condition factor (D), and HSI (E)) in saline–alkaline water for 50 days. All data are represented as the mean ± SEM (*n* = 3). Relationships of WG with dietary lipid levels (F) based on SOP regression analysis of *O. niloticus*, where Xopt represents the dietary lipid level (*n* = 3). Different letters in the same histogram show significant differences (*P*  < 0.05). The linear trend is indicated by L, while the adjusted *R*^2^ and second-order polynomial trend are represented by Adj. *R*^2^ and SOP, respectively. L3, C45%/L3%; L6, C38%/L6%; L9, C31%/L9%; L12, C24%/L12%; L15, C17%/L15%. ANOVA, analysis of variance; C, carbohydrate; FCR, feed conversion ratio; HSI, hepatosomatic index; L, lipid; SEM, standard error of the mean; WG, weight gain.

The growth performance of fish among groups is shown in Figure 1. There were no marked variations in SR, FCR, CF, or HSI between the different groups (*P* > 0.05, Figure 1A,C,D,E). However, the WG in the L12 group was markedly higher than that in the L3, L6, and L9 groups (*P* < 0.05, Figure 1B). In addition, the optimal ratio of dietary C–L was 2:1 (dry matter) by the quadratic broken line regression analysis of WG and C:L ratios (*P* < 0.05, Figure 1F).

### 3.2. Liver Histology

**Figure 2:**
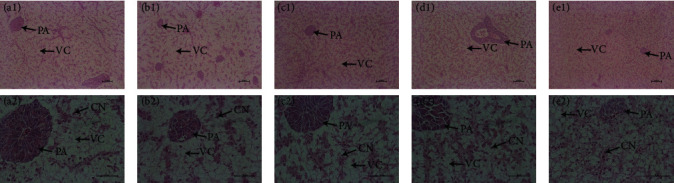
The effects of *O. niloticus* fed different diets on gill structure parameters in saline–alkaline water for 50 days. (A, a1 and a2) L3 group staining section of gill structure; (B, b1 and b2) L6 group staining section of gill structure; (C, c1 and c2) L9 group staining section of gill structure; (D, d1 and d2) L12 group staining section of gill structure; (E, e1 and e2) L15 group staining section of gill structure. (A–E), scale bar = 100 μm; (a–e), scale bar = 50 μm. C = carbohydrate; CN = cell nucleus; L = lipid; L3 = 3% L and 45% C; L6 = 6% L and 38% C; L9 = 9% L and 31% C; L12 = 12% L and 24% C; L15 = 15% L and 17% C; PA = pancreatic acini; VC = vacuolated cytoplasm.

The histopathological findings of the liver of fish in saline–alkali conditions are shown in Figure 2. The hepatocytes of all groups had clear edges, but the cell nuclei were shifted. Compared with the L9, L12, and L15 groups, disordered hepatic cords were observed in the L3 and L6 groups (Figure 2A,B (a and b)). In addition, although vacuolar degeneration of hepatocytes occurred in all groups, the proportion of vacuolar degeneration of hepatocytes decreased gradually with increasing dietary L content (Figure 2C,D, and E (c, d and e)).

### 3.3. C Metabolism

**Figure 3:**
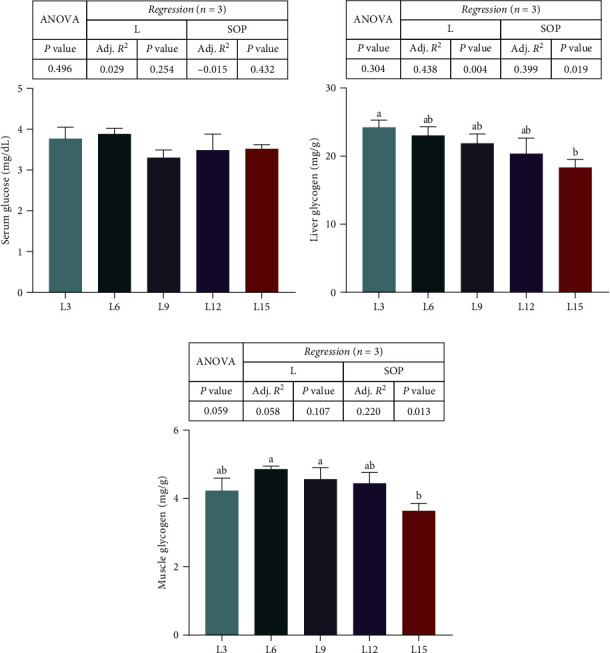
The effects of *O. niloticus* fed different diets on saccharides (serum glucose (A), liver glycogen (B), and muscle glycogen (C)) in saline–alkaline water for 50 days. All data are represented as the mean ± SEM (*n* = 3). Different letters in the same histogram show significant differences (*P*  < 0.05). The linear trend is indicated by L, while the adjusted *R*^2^ and second-order polynomial trend are represented by Adj. *R*^2^ and SOP. L3, C45%/L3%; L6, C38%/L6%; L9, C31%/L9%; L12, C24%/L12%; L15, C17%/L15%. ANOVA, analysis of variance; C, carbohydrate; L, lipid; SEM, standard error of the mean.

Systemic glycogen variations from treatments are shown in Figure 3. The high-C groups had higher serum glucose than the high-L groups, but the difference was not marked among groups (*P* > 0.05, Figure 3A,B). Additionally, the L3 group had higher liver glycogen levels than the L15 group (*P* < 0.05, Figure 3B), and the muscle glycogen in the L6 and L9 groups was significantly higher than that in the L15 group (*P* < 0.05, Figure 3C).

**Figure 4:**
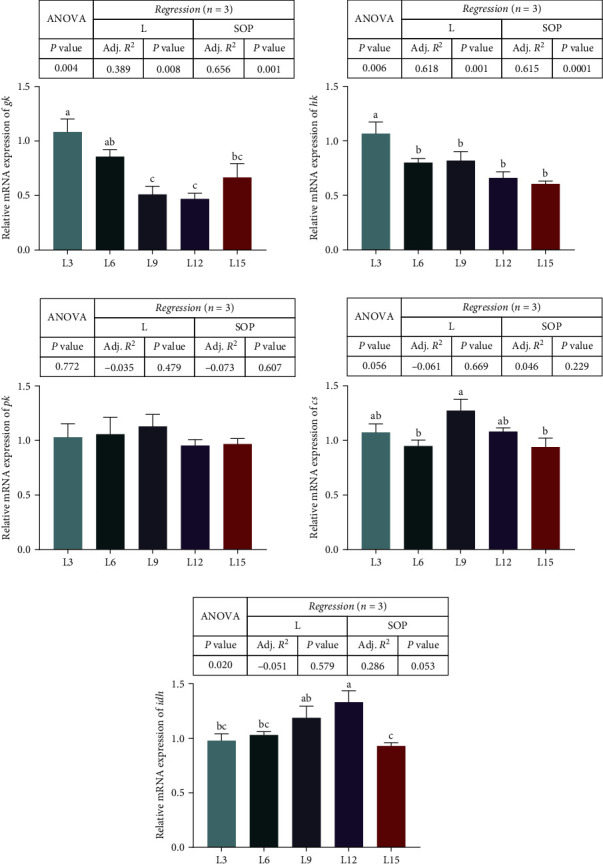
Expression of genes related to glycometabolism (*gk* (A), *hk* (B), *pk* (C), *cs* (D), and *idh* (E)) in the liver of *O. niloticus* fed different diets under saline–alkaline water for 50 days. Data are expressed as the mean ± SEM (*n* = 3). Different letters in the same histogram show significant differences (*P*  < 0.05). The linear trend is indicated by L, while the adjusted *R*^2^ and second-order polynomial trend are represented by Adj. *R*^2^ and SOP. L3, C45%/L3%; L6, C38%/L6%; L9, C31%/L9%; L12, C24%/L12%; L15, C17%/L15%. ANOVA, analysis of variance; C, carbohydrate; L, lipid; *cs*, citrate synthase; *gk*, glucokinase kinase; *hk*, hexokinase; *idh*, isocitrate dehydrogenase; *pk*, pyruvate kinase; SEM, standard error of the mean.

The relative mRNA expression of genes involved in glycometabolism is presented in Figure 4. The markedly highest mRNA expression of the glucokinase kinase (*gk*) and hexokinase (*hk*) genes was found in the L3 groups (*P* < 0.05, Figure 4A,B). Specifically, no variations were detected in the mRNA expression of the pyruvate kinase (*pk*) gene (*P* > 0.05, Figure 4C). For the tricarboxylic acid cycle, the L9 group had the highest expression of citrate synthase (*cs*) (*P* < 0.05, Figure 4D), and the L15 group had the highest expression of isocitrate dehydrogenase (*idh*; *P* < 0.05; Figure 4E).

### 3.4. Ion Transporters in Gills

**Figure 5:**
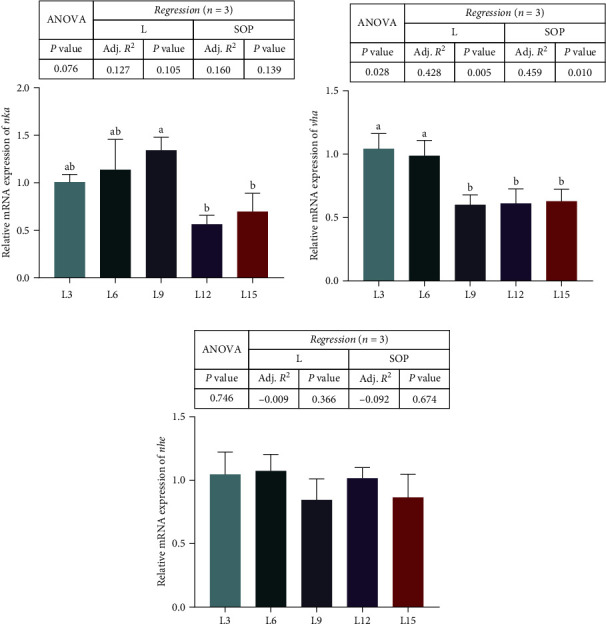
Expression of genes related to ion transporters (*nka* (A), *vha* (B), and *nhe* (C)) in the gills of *O. niloticus* fed different diets under saline–alkaline water for 50 days. Data are expressed as the mean ± SEM (*n* = 3). Different letters in the same histogram show significant differences (*P*  < 0.05). The linear trend is indicated by L, while the adjusted *R*^2^ and second-order polynomial trend are represented by Adj. *R*^2^ and SOP. L3, C45%/L3%; L6, C38%/L6%; L9, C31%/L9%; L12, C24%/L12%; L15, C17%/L15%. ANOVA, analysis of variance; C, carbohydrate; L, lipid; *nhe*, Na^+^/H^+^-exchanger; *nka*, Na^+^/K^+^-ATPase; SEM, standard error of the mean; *vha*, V/H^+^-ATPase.

The expression of genes associated with ion transporters is presented in Figure 5. The mRNA levels of Na^+^/K^+^-ATPase (*nka*) in the L9 group were markedly higher than those in the L12 and L15 groups (*P* < 0.05, Figure 5A). The mRNA expression of V/H^+^-ATPase (*vha*) was higher in the L3 and L6 groups than in the other groups (Figure 5B). However, there were no marked variations in Na^+^/H^+^-exchanger (*nhe*) among the groups (*P* > 0.05, Figure 5C).

### 3.5. L Metabolism

**Figure 6:**
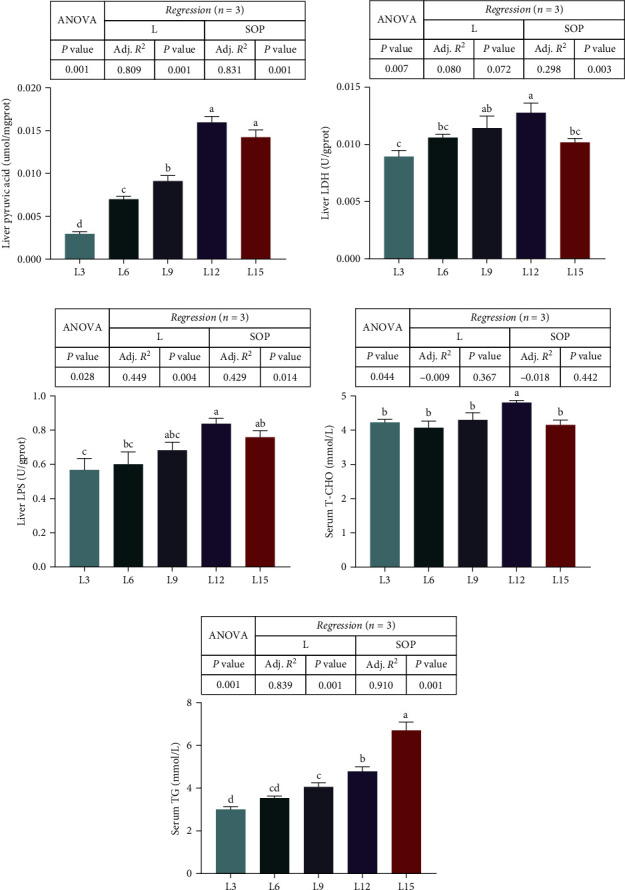
The effects of *O. niloticus* fed different diets on enzyme activities in different tissues (liver pyruvic acid (A), liver LDH (B), liver LPS (C), serum T-CHO (D), and serum TG (E)) under saline–alkaline stress for 50 days. All data are represented as the mean ± SEM (*n* = 3). Different letters in the same histogram show significant differences (*P*  < 0.05). The linear trend is indicated by L, while the adjusted *R*^2^ and second-order polynomial trend are represented by Adj. *R*^2^ and SOP. L3, C45%/L3%; L6, C38%/L6%; L9, C31%/L9%; L12, C24%/L12%; L15, C17%/L15%. ANOVA, analysis of variance; C, carbohydrate; L, lipid; LDH, lactic dehydrogenase; LPS, lipase; SEM, standard error of the mean; T-CHO, total cholesterol; TG, triglyceride.

Biochemical analysis of serum and liver is shown in Figure 6. The liver pyruvic acid increased significantly with increasing dietary Ls, and it reached a maximum in the L12 and L15 groups (*P* < 0.05, Figure 6A). Likewise, liver LDH, liver LPS, and serum T-CHO were highest in L12 (*P* < 0.05, Figure 6B,C,D). Nevertheless, the serum TG of the L15 group was markedly higher than that of the other groups (*P* < 0.05, Figure 6E).

**Figure 7:**
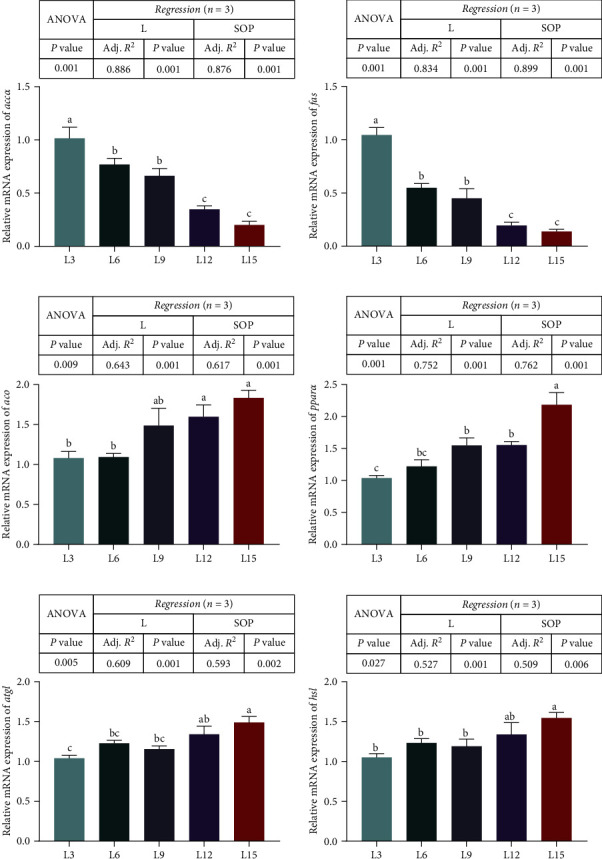
Expression of genes related to lipid metabolism (*accα* (A), *fas* (B), acyl-coenzyme (C), *pparα* (D), *atgl* (E), *hsl* (F)) in the liver of *O. niloticus* fed different diets under saline–alkaline water for 50 days. Data are expressed as the mean ± SEM (*n* = 3). Different letters in the same histogram show significant differences (*P*  < 0.05). The linear trend is indicated by L, while the adjusted *R*^2^ and second-order polynomial trend are represented by Adj. *R*^2^ and SOP. L3, C45%/L3%; L6, C38%/L6%; L9, C31%/L9%; L12, C24%/L12%; L15, C17%/L15%. *accα*, acetyl-CoA carboxylase *α*; *aco*, acyl-coenzyme An oxidase; Adj. *R*^2^, adjusted *R*^2^; ANOVA, analysis of variance; C, carbohydrate; *atgl*, adipose triglyceride lipase; *fas*, fatty acid synthase; *hsl*, hormone-sensitive lipase; L, lipid; *pparα*, peroxisome proliferator activated receptor *α*; SEM, standard error of the mean.

As shown in Figure 7, the L3 group had higher mRNA levels of acetyl-CoA carboxylase *α* (*accα*) and fatty acid synthase (*fas*) than the other groups (*P* < 0.05, Figure 7A,B). Nevertheless, the expression level of acyl-coenzyme An oxidase (*aco*) was significantly lower in the L3 and L6 groups than in the L12 and L15 groups (*P* < 0.05, Figure 7C). Specifically, the mRNA levels of peroxisome proliferator activated receptor *α* (*pparα*), adipose triglyceride lipase (*atgl*), and hormone-sensitive lipase (*hsl*) markedly increased with increasing dietary Ls and reached a maximum in the L15 group (*P* < 0.05, Figure 7D,E,F).

### 3.6. Serum Ammonia and Ammonia Metabolism

**Figure 8:**
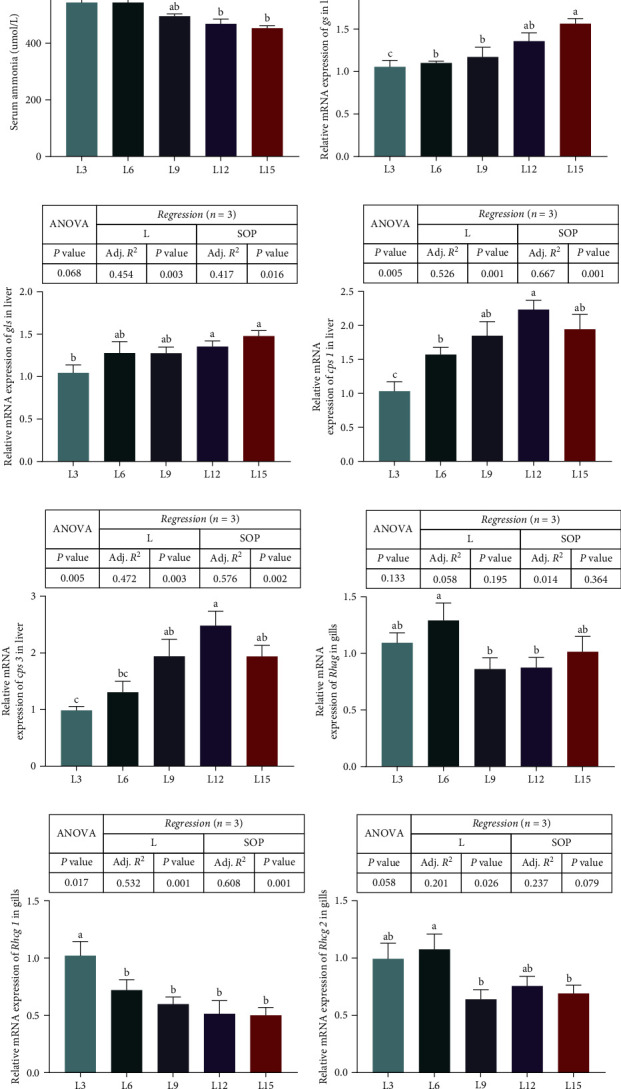
Serum ammonia (A) and expression of genes related to ammonia metabolism (*gs* (B), *gls* (C), *cps 1* (D), *cps* 3 (E), *Rhag* (F), *Rhcg 1* (G), and *Rhcg 2* (H)) in the different tissues of *O. niloticus* fed different diets under saline–alkaline water for 50 days. Data are expressed as the mean ± SEM (*n* = 3). Different letters in the same histogram show significant differences (*P*  < 0.05). The linear trend is indicated by L, while the adjusted R square and second-order polynomial trend are represented by Adj. R^2^ and SOP. L3, C45%/L3%; L6, C38%/L6%; L9, C31%/L9%; L12, C24%/L12%; L15, C17%/L15%. ANOVA, analysis of variance; C, carbohydrate; *cps 1*, carbamoyl phosphate synthase 1; *cps 3*, carbamoyl phosphate synthase 3; *gls*, glutaminase; *gs*, glutamine synthetase; L, lipid; *Rhag*, Rh type a; *Rhcg 1*, Rh type c1; *Rhcg 2*, Rh type c2; SEM, standard error of the mean.

The L3 and L6 groups had markedly higher serum ammonia levels than the L12 and L15 groups (*P* < 0.05, Figure 8A). The expression levels of glutamine synthetase (*gs*) and glutaminase (*gls*) markedly increased with increasing dietary Ls, and the expression level of *gs* reached a maximum in the L15 group (*P* < 0.05, Figure 8B), while the expression level of *gls* reached a maximum in the L12 and L15 groups (*P* < 0.05, Figure 8C). Analogously, the highest expression levels of carbamoyl phosphate synthase 1 (*cps 1*) and carbamoyl phosphate synthase 3 (*cps 3*) were found in the L12 groups (*P* < 0.05, Figure 8D,E). However, the L6 group had significantly higher mRNA levels of Rh type a (*Rhag*) and Rh type c2 (*Rhcg* 2; *P* < 0.05, Figure 8F,H). Higher expression of Rh type c1 (*Rhcg 1*) was found in the L3 group than in the other groups (*P* < 0.05, Figure 8G).

### 3.7. Association Between Growth Performance and Tissue Glycogen, Glycolipid Metabolism, and Ammonia Metabolism

**Figure 9:**
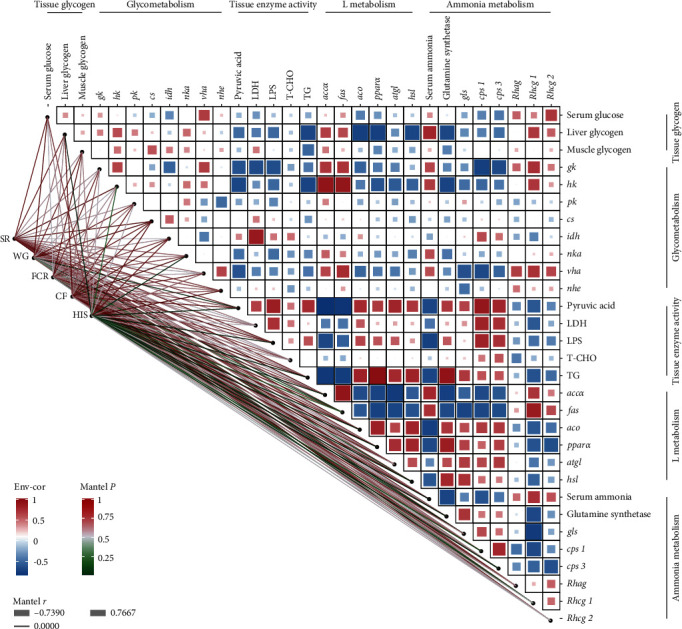
Correlations among growth performance, biochemical analysis, and quantitative RT-PCR. The edge width of lines refers to Mantel's *r* for the statistics of corresponding distance correlations, and the color of lines represents the statistical significance. RT-PCR, *accα*, acetyl-CoA carboxylase *α; aco, acyl-coenzyme An oxidase; atgl*, adipose triglyceride lipase; *cps 1*, carbamoyl phosphate synthase 1*; cps 3*, carbamoyl phosphate synthase 3; *cs*, citrate synthase; Env-cor, environmental correlation; *fas*, fatty acid synthase; *gk*, glucokinase kinase; *gls*, glutaminase; *hk*, hexokinase; *hsl*, hormone-sensitive lipase; *idh*, isocitrate dehydrogenase; LDH, lactic dehydrogenase; LPS, lipase; *nka*, Na^+^/K^+^-ATPase; *pk*, pyruvate kinase; *pparα*, peroxisome proliferator activated receptor alpha; *Rhag*, Rh type a; *Rhcg 1*, Rh type c1; *Rhcg 2*, Rh type c2; T-CHO, total cholesterol; TG, triglyceride; *vha*, V/H^+^-ATPase.

As shown in Figure 9, the growth performance of tilapia was closely related to tissue glycogen, glycolipid metabolism, and ammonia metabolism. The expression levels of Rh family genes associated with ammonia excretion in the gills were significantly positively correlated with glycolytic capacity and tissue glycogen content (*P* < 0.05). The expression levels of ammonia metabolism–related genes glutamine synthetase, *gls*, *cps 1*, and carbamoyl phosphate synthase 2 (*cps 2*) in the liver were significantly positively correlated with L metabolism and significantly negatively correlated with serum ammonia levels (*P* < 0.05). Additionally, the SR was not significantly correlated with tissue glycogen, glycolipid metabolism, and ammonia metabolism (*P* > 0.05). The FCR was significantly positively correlated with the expression levels of *hk* genes and LPS enzyme activity (*P* < 0.05). The HSI was significantly negatively correlated with the expression levels of *accα*, *aco*, and *pparα* genes (*P* < 0.05).

## 4. Discussion

Fish usually consume more energy when adapting to environmental stress [15]. In response to various environmental stresses, fish will adjust their energy metabolism to meet the demand for energy [39, 40]. Protein is a vital nutrient for the growth and development of fish [41]. However, studies have shown that increasing dietary protein levels can negatively affect the growth and immune function of *Cyprinus carpio Songpu* under alkalinity stress [19]. Cs and Ls both have protein-sparing effects and are good sources of energy [42, 43]. The findings of the present study indicate that the dietary carbohydrate-lipidC:L ratios did not affect the SR, FCR, or CF of tilapia under saline–alkali stress. Specifically, excessive or insufficient dietary Cs and Ls can disrupt energy metabolism and damage liver health, thereby, reducing the growth of fish [17, 44]. This result found that the L12 group had a better WG than the other groups, indicating that the L12 diet may be more beneficial to the energy supply of tilapia in saline–alkali water. Taking the value of the WG as the evaluation standard of growth performance, our study found that the ideal dietary C:L ratio of tilapia under saline–alkali stress was 2:1. HSI is considered to be the ultimate indicator of L deposition in fish [45]. Usually, either a high-L diet or a high-C diet can cause L deposition in fish [21, 43, 46]. However, research has demonstrated that the accumulation of liver glycogen in tilapia caused by a high-C diet was more significant than that caused by a high-L diet, which led to a higher HSI [47]. Additionally, previous studies have shown that fish fed high-L diets can reduce liver enlargement and cytoplasmic vacuolation compared to fish fed high-C diets [47–49]. In this study, the liver tissue histology showed that all groups exhibited some hepatocyte vacuolar degeneration due to saline–alkali stress, and the proportion of hepatocyte vacuolar degeneration and disordered hepatic cords decreased gradually with the increase in dietary L levels. Therefore, this study suggested that the high-L diet was more conducive to the liver health of tilapia than the high-C diet under saline–alkali stress, which aligns with the findings found in fresh water [17]. In addition, the results of association analysis in this study also showed that HSI was significantly negatively correlated with the level of gene expression associated with L metabolism. In conclusion, appropriately increasing the proportion of dietary Ls may be more conducive to the WG and liver health of tilapia under saline–alkali stress.

Cs are used as one of the main components of fish diets because of their easy availability, low price, and excellent energy supply [50]. Previous studies have shown that the serum glucose and tissue glycogen levels of tilapia increase with increasing dietary C levels [47, 51]. This is because a C-rich diet can promote insulin production, which in turn increases glycogen levels to maintain glucose balance [52]. Our study also found that tilapia fed high-C diets were more likely to accumulate liver glycogen in saline–alkali water. Some studies have demonstrated that fish can use glycogen as an energy source for osmoregulation [9, 53], and high-C diets have been proven to provide additional energy for osmotic regulation of euryhaline fish [26, 54, 55]. To date, some scholars have found that glucose metabolism in aquatic animals is closely related to tolerance to saline–alkali stress [6, 56]. Similarly, this study found that the fish fed high-C diets enhanced the expression of crucial genes associated with glycolytic reactions, such as *gk* and *hk*, in the liver compared with those fed high-L diets under saline–alkali stress. This indicated that dietary Cs play an important role in the energy supply of tilapia in saline–alkali water. As the source of essential fatty acids, dietary Ls also have a significant impact on energy provision and the regulation of L metabolism [27]. Research has indicated that maintaining stable L metabolism can assist aquatic animals in adapting to environmental stresses, such as salinity, hypoxia, and ammonia poisoning [57–59]. In fish, the stability of L metabolism is mainly affected by L uptake, transport, secretion, lipogenesis, and lipolysis [59, 60]. In general, the expression of the *accα* and *fas* genes involved in L synthesis increased significantly with decreasing dietary Ls [17], and similar results appeared in this test. This phenomenon occurs because fish consuming high-C diets with low-L can convert excess Cs into Ls through lipogenesis, thereby [61, 62]. This result is broadly supported by other nutritional tests related to aquatic animals, such as rainbow trout (*Oncorhynchus mykiss*) [63] and large yellow croaker (*Larimichthys crocea*) [62]. As reported, metabolomics analysis in some studies showed that L metabolism could provide much energy and essential fatty acids for aquatic animals to adapt to saline–alkali water [6, 56]. Lipohydrolysis and *β*-oxidation are important ways of supplying energy to aquatic animals [64, 65]. Studies have shown that the lipohydrolysis and *β*-oxidation of fish in freshwater decreases with increasing dietary Cs [62]. In this study, the fish fed high-C diets had decreased expression of key genes associated with lipohydrolysis and *β*-oxidation, such as *aco*, *pparα*, *atgl*, and *hsl*, in the liver compared with those fed high-L diets, which meant that dietary Ls also played an important role in providing energy for tilapia to cope with saline–alkali stress. However, it should be noted that excessive dietary Ls can destroy the stability of L metabolism in fish [66]. The results of this research showed that the highest liver LPS activities and serum T-CHO concentrations were found in the L12 group. LPS is recognized as a key enzyme in L uptake [17], and T-CHO is commonly employed as a measure of endogenous L transport activity [44]. Therefore, the results of this study indicated that 15% dietary Ls can disrupt normal L metabolism by decreasing L uptake and L transport. In general, dietary Ls were one of the energy sources of tilapia under saline–alkali stress, and 12% dietary Ls may be more conducive to L homeostasis. In general, dietary Ls were one of the energy sources of tilapia under saline–alkali stress, and 12% dietary Ls may be more conducive to L homeostasis.

C metabolism and L metabolism are not only closely related to energy metabolism [67, 68], but also affect osmotic regulation and ammonia metabolism [58, 69, 70]. It is well known that Cs play a direct role in the energy supply for osmotic regulation caused by salinity stress [71, 72]. In addition, research findings have indicated that hard clams (*Mercenaria mercenaria*) preferentially use glucose metabolism to provide energy for osmotic regulation [70]. C catabolism is the main source of energy for fish to cope with salinity stress [73]. This might be the reason why tilapia fed a high-C diet showed better ion transport performance than tilapia fed a high-L diet in this study. Both alkalinity stress and salinity stress can hinder the excretion of ammonia and cause osmotic imbalance in aquatic animals [11, 12, 74]. Some scholars believe that the most important thing for aquatic animals adapting to saline–alkali stress is to alleviate ammonia poisoning [75, 76]. Therefore, many fish living in saline–alkaline water have evolved corresponding ammonia excretion mechanisms [77, 78]. Some fish can use their gills to release ammonia through proteins such as Rh glycoproteins located on the basal and top plasma membranes of branchial squamous cells [77, 79]. In the meantime, some blood ammonia in the body could be synthesized into glutamine, which is an important detoxification method for some fish to reduce ammonia accumulation [80, 81]. In addition, some ammonia could also be synthesized into urea, which is a strategy used by a few fish species for ammonia detoxification [16]. Ammonia is usually excreted as NH_3_ from fish gills into the surrounding water [82]. Some studies have proven that Rh glycoprotein transportation is an important mechanism of ammonia excretion in fish gills [83, 84]. In this study, tilapia fed high-C diets had a higher gene expression level of Rh glycoproteins than those fed high-L diets. This is because the ammonia excretion function of Rh glycoproteins is closely related to ion transport, such as Na^+^ and H^+^ [82, 85]. However, the serum ammonia levels of tilapia fed the high-C diets were markedly higher compared to those fed the high-L diets in saline–alkali water. This may be because the amount of ammonia excreted through the gills is less than the accumulation of ammonia caused by saline–alkali stress [70, 86, 87]. Therefore, we speculated that the metabolism of glutamine and urea in the liver might be the main strategy for tilapia to excrete ammonia in saline–alkali water. Fish can use ammonia and glutamate to synthesize nontoxic glutamine by the catalytic action of *gs* in the stored liver [88]. Then, glutamine is decomposed back into ammonia by the action of *gls* in gills, after which ammonia is excreted out of the body with the help of transport proteins [89]. Tilapia fed high-L diets had a higher gene expression level of *gs* and *gls* than those fed high-C diets in this study. In addition, as the rate-limiting enzyme of the ornithine cycle, *cps* can synthesize urea from ammonia, which is then excreted from the body [90]. A higher gene expression level of cps was observed in the high-L groups in this study. In conclusion, combined with glutamine and urea metabolism and serum ammonia in this study, we speculated that dietary Ls were more conducive to ammonia excretion of tilapia in saline–alkali water. The results of association analysis also showed that the serum ammonia level was negatively correlated with the expression level of L metabolism–related genes in tilapia. However, there are few studies on the relationship between dietary Ls and glutamine and urea metabolism, and further studies are needed.

## 5. Conclusion

This study showed that both Cs and Ls were important energy sources of tilapia under saline–alkali stress. Cs could help tilapia resist osmotic regulation caused by saline–alkali stress in gills, while L metabolism is more conducive to ammonia metabolism in tilapia under saline–alkali stress in livers. Specifically, tilapia fed high-C diets had higher expression of genes involved in ammonia transporters, and tilapia fed high-L diets had higher expression of genes associated with glutamine metabolism and urea metabolism. However, the tilapia fed high-L diets had lower serum ammonia levels than those fed high-C diets. Therefore, glutamine metabolism and urea metabolism were the main coping strategies for tilapia to cope with the burden of ammonia metabolism caused by saline–alkali stress. This finding holds significant implications for the breeding of saline–alkali tolerant species and the development of corresponding feeds. Moreover, the dietary C:L ratio of 2:1 was more conducive to WG in tilapia under saline–alkali stress. This study not only provided a new nutrient regulation strategy for saline–alkali water culture but also clarified the corresponding metabolic mechanism of tilapia in saline–alkali water and provided a new idea for the development and utilization of saline–alkali water.

## Figures and Tables

**Table 1:** Formulation and chemical composition of experimental diets (as percent of dry matter).

Ingredients	Diets				
	L3	L6	L9	L12	L15
Fish meal (729 g/kg protein)	10	10	10	10	10
Soybean meal (498 g/kg protein)	8	8	8	8	8
Corn meal(632 g/kg protein)	29	29	29	29	29
Corn oil	2	5	8	11	14
Corn starch	45	38	31	24	17
Cellulose	0.8	4.8	8.8	12.8	16.8
Vitamin mix^1^	2	2	2	2	2
Mineral mix^2^	2	2	2	2	2
Ca(H_2_PO_4_)_2_ Choline chloride	1 0.2	1 0.2	1 0.2	1 0.2	1 0.2
Total	100	100	100	100	100
Chemical composition (%)					
Moisture	9.25	8.84	8.67	8.30	7.90
Crude protein	32.78	32.25	32.23	32.57	33.56
Crude lipid	3.50	5.98	9.01	11.81	14.17
Ash	5.67	5.76	5.60	5.52	5.82
Total energy (kJ/g)^3^	16.86	16.51	16.50	16.48	16.44

Abbreviations: C, carbohydrate; L, lipid; L3, 3% L and 45% C; L6, 6% L and 38% C; L9, 9% L and 31% C; L12, 12% L and 24% C; L15, 15% L and 17% C.

^1^Vitamin premix (mg/kg): vitamin A (500,000 IU/g), 8 mg; vitamin D3 (1,000,000 IU/g), 2 mg; vitamin K, 10 mg; vitamin E, 200 mg; thiamine, 10 mg; riboflavin, 12 mg; pyridoxine, 10 mg; calcium pantothenate, 32 mg; nicotinic acid, 80 mg; folic acid, 2 mg; vitamin B12, 0.01 mg; biotin, 0.2 mg; choline chloride, 400 mg; vitamin C-2- polyphosphate (150 mg/g vitamin C activity), 400 mg.

^2^Mineral premix (mg/kg): zinc (ZnSO_4_·7H_2_O), 50.0 mg; iron (FeSO4·7H_2_O), 40 mg; manganese (MnSO_4_·7H_2_O), 15.3 mg; copper (CuCl_2_), 3.8 mg; iodine (KI), 5 mg; cobalt (CoCl_2_·6H_2_O), 0.05 mg; selenium (Na_2_SeO_3_), 0.09 mg. c *β*-Glucan in this study was extracted from yeast cell walls and purchased from Swiss ABAC. Research and Development Co., Ltd. (Barden, Swiss).

^3^Calculated values based on 23.6, 39.5, and 17.2 kJ/g for protein, lipid, and nitrogen-free extracts, respectively [35].

**Table 2:** List of the *O. niloticus* primers used for qRT-PCR.

Gene	Forward primer (5′ to 3′)	Reverse primer (5′ to 3′)	Product size (bp)	GenBank
*β-actin*	AGCCTTCCTTCCTTGGTATGGAAT	TGTTGGCGTACAGGTCCTTACG	102	KJ126772.1
*gk*	CAAGGGAATCCTGCTTAACTG	GAAATCATCGTGGCAACTGT	156	XM_003451020.2
*hk*	TTCCTCTGGGCTTCACCTTCT	ATCTTCCCCTTCGCAGTCTGT	109	XM_039611531.1
*pk*	CAGCATAATCTGCACCATCGGT	ATGAGAGAAGTTAAGACGGGCGA	100	XM_005472621.3
*cs*	CGACTGGTCCCACAACTTCA	GAGCACTAACATTGCCTCCTT	116	XM_003438897
*idh*	ACGCATCGCTGAGTACGCCTT	AGACCGTCTGACATCCGCATGA	99	XM_003437590.5
*nka*	CGTGCTGAATTTAAGGCAGGTCA	GCAAAGCTGATTCAGAAGCGTCAC	103	LC556924.1
*vha*	GACCATTCGCTGTCGTAAAC	CCAGCGGTATGTCCTGTTTA	237	AB369668.1
*nhe*	ATGAAGCGTCAGCCTAGGAA	TCCCAGAGCCTGGATCATAC	99	XM_003447282.5
*accα*	TAGCTGAAGAGGAGGGTGCAAGA	AACCTCTGGATTGGCTTGAACA	110	XM_005471970
*fas*	TCATCCAGCAGTTCACTGGCATT	TGATTAGGTCCACGGCCACA	102	GU433188
*aco*	AGTCCCACTGTGAGCTCCATCAA	CAGACCATGGCAGTTTCCAAGA	108	KF918710
*pparα*	GTTCCTCAAGAGTCTCCGCC	AAAGAGCTAGGTCGCTGTCG	107	KF871430
*atgl*	GACACATGCTGCAAAGCACT	ACCAGGACGTTTTCTCCGTC	103	XM_003440346.5
*hsl*	AGTTCACTCCAGCCATTCGG	TGGCTGCTACCCCTATTCCT	105	XM_005463937.4
*gs*	CGATCCATTCCGCAAAGA	ACAGGTGAGCCGAAGGTTG	94	AF503208.1
*gls*	CTTTTGCTCCAATGACTCTA	CTGCGATGGTGTAGAACAG	104	XM_025902817.1
*cps 1*	GGTCAGGCTGGTGAGTTT	ACAGAGGCTATGTTTGGATT	101	XM_003445297.5
*cps 3*	TGGGATTCAGGATTCGTTC	AGTGGCTTCAGTAGCATAGAG	91	MT119353.1
*Rhag*	CACCCACAATAACACTGACC	GAGCAAGTTGATGCCCAC	148	XM_003446300.4
*Rhcg 1*	TTTGGGATTACACTGTTTGC	CAAGTTAGGACGATAGAGCAT	144	MW448159.1
*Rhcg 2*	GCTTCATAGGTGCCATAGTT	CCCTGCGTTGACATAGTC	118	MW448160.1

Abbreviations: *accα*, acetyl-CoA carboxylase *α; aco, acyl-coenzyme An oxidase; atgl*, adipose triglyceride lipase; *cps 1*, carbamoyl phosphate synthase 1*; cps 3*, carbamoyl phosphate synthase 3; *cs*, citrate synthase; *fas*, fatty acid synthase; *gk*, glucokinase kinase; *gls*, glutaminase; *gs*, glutamine synthetase; *hk*, hexokinase; *hsl*, hormone-sensitive lipase; *idh*, isocitrate dehydrogenase; *nhe*, Na^+^/H^+^-exchanger; *nka*, Na^+^/K^+^-ATPase; *pk*, pyruvate kinase; *pparα*, peroxisome proliferator activated receptor alpha; *Rhag*, Rh type a; *Rhcg 1*, Rh type c1; *Rhcg 2*, Rh type c2; *vha*, V/H^+^-ATPase.

## Data Availability

The data can be available from the corresponding author upon reasonable request.
